# 2-Amino-5-methyl-3-(2-oxo-2-phenyl­eth­yl)-7-phenyl-4,5,6,7-tetra­hydro-3*H*-[1,2,4]triazolo[1,5-*a*]pyrimidin-8-ium bromide ethanol monosolvate

**DOI:** 10.1107/S1600536813025853

**Published:** 2013-09-25

**Authors:** Victor M. Chernyshev, Dmitriy A. Pyatakov, Kyrill Yu. Suponitsky

**Affiliations:** a South-Russia State Technical University, 346428 Novocherkassk, Russian Federation; b A. N. Nesmeyanov Institute of Organoelement Compounds, 119991 Moscow, Russian Federation

## Abstract

In the title compound, C_20_H_22_N_5_O^+^·Br^−^·C_2_H_6_O, the tetra­hydro­pyrimidine ring of the bicyclic cation adopts a half-chair conformation with an equatorial orientation of the phenyl and methyl substituents. The amino group is nearly coplanar with the 1,2,4-triazole ring [interplanar angle = 4.08 (8)°] and has a slightly pyramidal configuration. The mean planes of the triazole ring and the benzene ring of the phenacyl group form a dihedral angle of 88.58 (7)°. In the crystal, N—H⋯Br, N—H⋯O and O—H⋯Br hydrogen bonds link the cations, anions and ethanol mol­ecules into layers parallel to the *bc* plane.

## Related literature

For the synthesis and reactivity of partially hydrogenated [1,2,4]triazolo[1,5-*a*]pyrimidines, see: Desenko (1995 ▶); Desenko *et al.* (1994 ▶); Chebanov *et al.* (2008 ▶, 2010 ▶); Gorobets *et al.* (2012 ▶); Lipson *et al.* (2012 ▶), Chernyshev *et al.* (2008*a* ▶,*b* ▶). For applications of partially hydrogenated triazolo­pyrimidines in the synthesis of polycondensed heterocycles, see: Beck *et al.* (2011 ▶); Lipson *et al.* (2006 ▶); Sidorenko & Orlov (2011 ▶); Sokolov *et al.* (2011 ▶). For structures of protonated *C*-amino-1,2,4-triazoles and quaternized derivatives of tetra­hydro­triazolo­pyrimidines, see: Chernyshev *et al.* (2008*a* ▶, 2010 ▶); Matulkova *et al.* (2012 ▶). For standard bond lengths, see: Allen *et al.* (1987 ▶). For the correlation of bond lengths with bond orders between the *sp* ^2^ hybridized C and N atoms, see: Burke-Laing & Laing (1976 ▶). For puckering parameters, see: Cremer & Pople (1975 ▶).
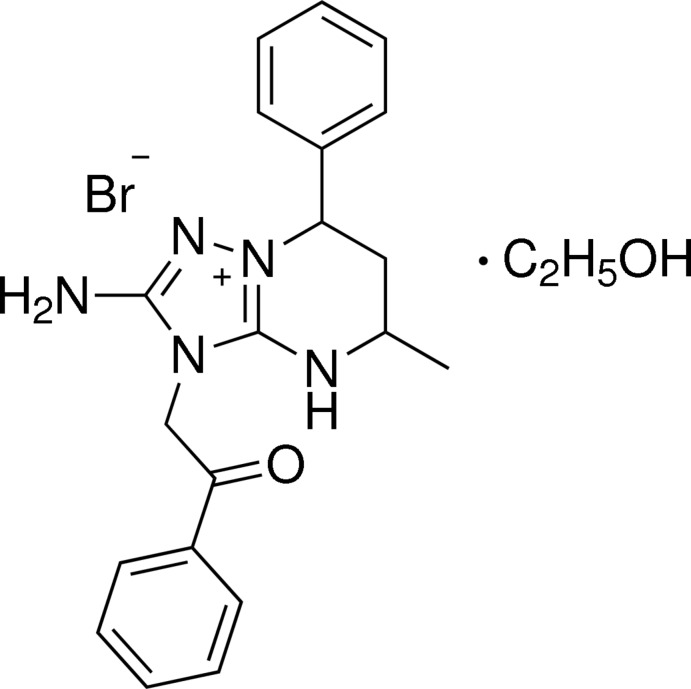


## Experimental

### 

#### Crystal data

C_20_H_22_N_5_O^+^·Br^−^·C_2_H_6_O
*M* *_r_* = 474.40
Monoclinic, 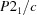

*a* = 10.7471 (13) Å
*b* = 13.3261 (16) Å
*c* = 15.5792 (19) Å
β = 95.735 (2)°
*V* = 2220.0 (5) Å^3^
*Z* = 4
Mo *K*α radiation
μ = 1.88 mm^−1^
*T* = 120 K
0.22 × 0.19 × 0.18 mm

#### Data collection

Bruker SMART APEXII CCD area-detector diffractometer
Absorption correction: multi-scan (*SADABS*; Bruker, 2008 ▶) *T*_min_ = 0.520, *T*_max_ = 0.713
31509 measured reflections
5356 independent reflections
4309 reflections with *I* > 2σ(*I*)
*R*_int_ = 0.054

#### Refinement

*R*[*F* ^2^ > 2σ(*F* ^2^)] = 0.032
*wR*(*F* ^2^) = 0.082
*S* = 1.01
5356 reflections
273 parameters
H-atom parameters constrained
Δρ_max_ = 0.87 e Å^−3^
Δρ_min_ = −0.48 e Å^−3^

### 

Data collection: *APEX2* (Bruker, 2009 ▶); cell refinement: *SAINT* (Bruker, 2009 ▶); data reduction: *SAINT*; program(s) used to solve structure: *SHELXTL* (Sheldrick, 2008 ▶); program(s) used to refine structure: *SHELXTL*; molecular graphics: *SHELXTL*; software used to prepare material for publication: *SHELXTL*, *publCIF* (Westrip, 2010 ▶) and *PLATON* (Spek, 2009 ▶).

## Supplementary Material

Crystal structure: contains datablock(s) I, New_Global_Publ_Block. DOI: 10.1107/S1600536813025853/aa2095sup1.cif

Structure factors: contains datablock(s) I. DOI: 10.1107/S1600536813025853/aa2095Isup2.hkl

Supplementary material file. DOI: 10.1107/S1600536813025853/aa2095Isup3.cdx

Supplementary material file. DOI: 10.1107/S1600536813025853/aa2095Isup4.cml

Additional supplementary materials:  crystallographic information; 3D view; checkCIF report

## Figures and Tables

**Table 1:** Hydrogen-bond geometry (Å, °)

*D*—H⋯*A*	*D*—H	H⋯*A*	*D*⋯*A*	*D*—H⋯*A*
N4—H3*N*⋯Br1^i^	0.90	2.49	3.392 (2)	176
N5—H1*N*⋯O1*S*	0.90	1.94	2.839 (2)	176
N5—H2*N*⋯Br1^ii^	0.90	2.61	3.468 (2)	159
O1*S*—H1*S*⋯Br1	0.85	2.46	3.287 (2)	165

Symmetry codes: (i) 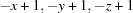; (ii) 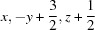.

**Fig. 1.**
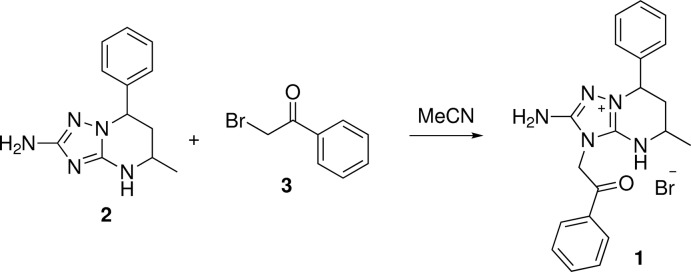
Synthesis of the title compound.

**Fig. 2.**
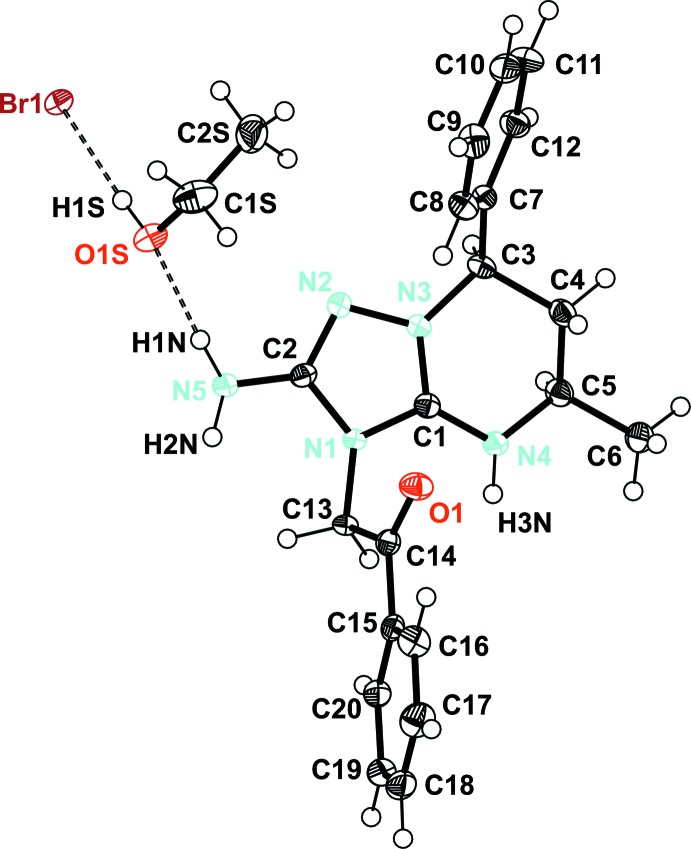
General view of the structure of the title compound with the atom numbering scheme. Displacement ellipsoids are drawn at the 50% probability level.

**Fig. 3.**
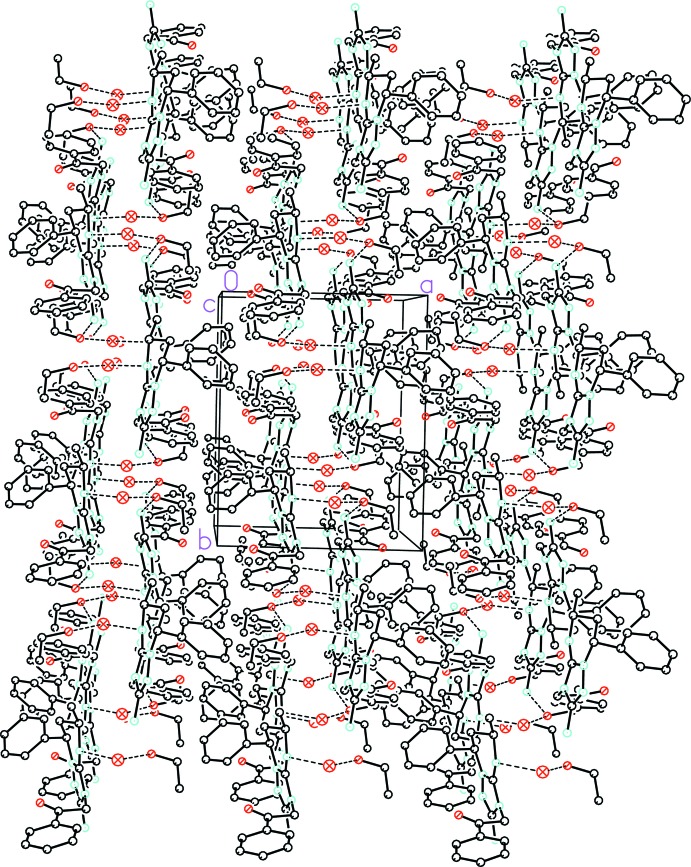
Crystal packing fragment showing layered structure of the title compound. Projection onto ab crystallographic plane.

## supplementary crystallographic information

### 1. Comment

In the last years much attention has been paid to the development of new methods for synthesis and investigation of properties of partially hydrogenated [1,2,4]triazolo[1,5-*a*]pyrimidines with various degree of saturation of pyrimidine ring (Chebanov *et al.*, 2008; Chebanov *et al.*, 2010; Gorobets *et al.*, 2012). In large part, the reason for this is that partially hydrogenated [1,2,4]triazolo[1,5-*a*]pyrimidines are the polyfunctional compounds capable to react with various electrophiles (Desenko, 1995; Chernyshev *et al.*, 2008*b*; Gorobets *et al.*, 2012; Lipson *et al.*, 2012). This capability can serve for the molecular design of triazolopyrimidine scaffold and construction of more complex heterocyclic systems. Thus, the 4,7-dihydro[1,2,4]triazolo[1,5-*a*]pyrimidines have received successful application for the synthesis of new polycondensed heterocycles (Desenko, 1995; Beck *et al.*, 2011; Lipson *et al.*, 2006; Sidorenko & Orlov, 2011). However, potential of 4,5,6,7-tetrahydro[1,2,4]triazolo[1,5-*a*]pyrimidines for synthesis of polycondensed heterocycles remain almost unexplored. We have assumed the possibility of application of the tetrahydrotriazolopyrimidines as new *N*,*N*-binucleophilic synthons for preparation of polycondensed heterocycles. Recently, by the example of reaction of 2-amino-substituted 4,5,6,7-tetrahydro[1,2,4]triazolo[1,5-*a*]pyrimidines with 3-chloropropanoic acid chloride, we have reported a simple method of preparation of 8-oxo-1,2,3,4,7,8,9,10-octahydro[1,2,4]triazolo[1,5 - *a*:4,3-*a'*]dipyrimidin-5-ium chlorides (Sokolov *et al.*, 2011). Continuing our work on the elaboration of new methods for synthesis of polycondensed heterocycles, we investigated the reaction of 2-amino-substituted 4,5,6,7-tetrahydro[1,2,4]triazolo[1,5-*a*]pyrimidines with α-bromoketones as potential source of imidazotriazolopyrimidines (the detailed results will be published later). The present article reports the molecular and crystal structures of compound 1, the product of reaction between 5-methyl-7-phenyl-4,5,6,7-tetrahydro[1,2,4]triazolo[1,5-*a*]pyrimidin-2-amine (2) and 2-bromo-1-phenylethanone (3) in acetonitrile (Fig. 1).

In accordance with the X-ray diffraction data (Fig. 2), the triazole ring (C1/N1/C2/N2/N3) is planar, with the mean deviations of the ring atoms from their least-squares plane being 0.014 (2) Å. In analogy with the previously described salts of *C*-amino-1,2,4-triazoles (Chernyshev *et al.*, 2010; Matulkova *et al.*, 2012) and quaternized derivatives of tetrahydrotriazolopyrimidines (Chernyshev *et al.*, 2008*a*) the bonds C1—N3 (1.323 (2) Å) and C2—N2 (1.312 (2) Å) are shorter than the bonds C1—N1 (1.362 (2) Å) and C2—N1 (1.394 (2) Å). The atoms N4 and N5 adopt a trigonal pyramidal configuration (the sums of valence angles are 349.8° and 357.2°, correspondingly) and deviates from the triazole plane by only 0.005 (2) Å and -0.020 (2) Å, respectively. Conjugation between the unshared electron pairs of N4 and N5 with the π–system of the triazole fragment leads to a shortening of the bonds C1—N4 (1.337 (2) Å) and C2—N5 (1.347 (2) Å) relative to the standard length of a purely single N*sp^2^*–C*sp*^2^ bond (1.43–1.45 Å) (Burke-Laing & Laing, 1976; Allen *et al.*, 1987). The tetrahydropyrimidine ring (C1/N3/C3/C4/C5/N4) adopts distorted half-chair conformation which was observed in other tetrahydro[1,2,4]triazolo[1,5-*a*]pyrimidines (Desenko *et al.*, 1994), the Cremer-Pople ring puckering coordinates (Cremer & Pople, 1975) calculated with the help of *PLATON* program (Spek, 2009) are: *Q* = 0.470 (2) Å, *Θ* = 132.5 (2)°, *φ* = 38.1 (3)°. The phenyl and methyl substituents have equatorial orientation. Bond lengths and angles in the phenacyl group are within the normal ranges. Carbonyl group is slightly noncoplanar to the benzene ring, torsion angle O1—C14—C15—C16 is 13.9 (3)°. Dihedral angle between the mean square planes of triazole cycle and carbonyl group (O1/C13/C14/C15) is 77.41 (8)°.

The title compound contains four acidic hydrogen atoms at the NH_2_, NH and OH groups which all participate in H-bonding. Three H-bonds are formed with the bromine anion and one with the oxygen atom of the hydroxyl group. However the carbonyl oxygen atom which is known to be a strong proton-acceptor is not involved in H-bonding. Four H-bonds listed in Table 1 leads to formation of H-bonded layers parallel to *bc* crystallographic plane. All the other intermolecular contacts correspond to the ordinary van-der-Waals interactions.

### 2. Experimental

The crystals of the title compound suitable for X-ray analysis were grown by slow evaporation of ethanol solution of 2-amino-5-methyl-3-(2-oxo-2-phenylethyl)-7-phenyl-4,5,6,7-tetrahydro-3*H*-[1,2,4]triazolo[1,5-*a*]pyrimidin-8-ium bromide (1) at room temperature within a week. The compound 1 was prepared by the following procedure.

A mixture of 5-methyl-7-phenyl-4,5,6,7-tetrahydro[1,2,4]triazolo[1,5-*a*]pyrimidin-2-amine (2, 0.459 g, 2 mmol), 2-bromo-1-phenylethanone (3, 0.597 g, 3.0 mmol) and acetonitrile (4 ml) was refluxed for 3 h, then evaporated to a volume of about 2 ml and cooled to 0 °C. The precipitate formed was isolated by filtration, recrystallized from ethanol and dried at 100 °C to give 0.732 g (57% yield) of white powder, m. p. 190–192 °C. Spectrum ^1^H NMR (500 MHz), *d:* 1.24 (d' *J* = 5.7 Hz, 3H, Me), 1.90–1.95 (m, 1H, H-6a), 2.43–2.45 (m, 1H, H-6 b), 3.82–3.85 (m, 1H, H-5), 5.25 (dd, *J* = 10.9,4.9 Hz, 1H, H-7), 5.54 (s, 2H, NCH_2)_, 6.69 (s, 2H, NH_2_), 7.34–7.44 (m, 5H, Ar), 7.65–7.67 (m, 3H, Ar), 7.77–7.79 (m, 1H, Ar), 8.08–8.09 (m, 1H, Ar), 8.70 (s, 1H, NH). Spectrum ^13^C NMR (125 MHz), *d*: 19.55 (Me), 38.65 (C-6), 45.99 (C-5), 48.80 (NCH_2_), 58.45 (C-7), 127.52, 128.28, 128.31, 128.53, 128.92, 133.87, 134.32, 137.77 (carbons of two benzene rings), 146.52 (C-3a), 149.96 (C-2), 190.33 (CO). MS (EI, 70 eV), *m*/*z* (%):348 (2) [*M* – Br]^+^, 347 (10) [*M* – HBr]^+^, 332 (21), 229 (13), 201 (11), 131 (14), 125 (17), 115 (11), 105 (100), 100 (26), 91 (52), 82 (26), 80 (27), 77 (89), 65 (13), 51 (38), 43 (36). Anal. Calcd for C_20_H_22_BrN_5_O: C 56.08; H 5.18; N 16.35. Found: C 56.23; H 5.34; N 16.04.

The starting compound 2 was prepared by known method (Chernyshev *et al.*, 2008*b*).

### 3. Refinement

The hydrogen atoms of NH, NH_2_ and OH groups were found in difference Fourier synthesis and normalized to the standard X-ray value of 0.90 Å for NH and NH_2_ groups, and 0.85 Å for OH group. The H(C) atom positions were calculated. All the hydrogen atoms were refined in isotropic approximation in riding model with the *U*_iso_(H) parameters equal to 1.5 *U*_eq_(Ci), 1.2 *U*_eq_(Cj), 1.2 *U*_eq_(N), 1.5 *U*_eq_(O) where *U*_eq_(Ci) and *U*_eq_(Cj) are the equivalent thermal parameters of the methyl carbon atoms and all the other carbon atoms, respectively, to which corresponding H atoms are bonded; *U*_eq_(N) and *U*_eq_(O) are the equivalent thermal parameters of the nitrogen and oxygen atoms, respectively, to which corresponding H atoms are bonded.

### Crystal data

**Table:**

C_20_H_22_N_5_O^+^·Br^−^·C_2_H_6_O	*F*(000) = 984
*M**_r_* = 474.40	*D*_x_ = 1.419 Mg m^−^^3^
Monoclinic, *P*2_1_/*c*	Melting point: 190 K
Hall symbol: -P 2ybc	Mo *K*α radiation, λ = 0.71073 Å
*a* = 10.7471 (13) Å	Cell parameters from 8717 reflections
*b* = 13.3261 (16) Å	θ = 2.4–28.0°
*c* = 15.5792 (19) Å	µ = 1.88 mm^−^^1^
β = 95.735 (2)°	*T* = 120 K
*V* = 2220.0 (5) Å^3^	Prizm, colourless
*Z* = 4	0.22 × 0.19 × 0.18 mm

### Data collection

**Table:**

Bruker SMART APEXII CCD area-detector diffractometer	5356 independent reflections
Radiation source: fine-focus sealed tube	4309 reflections with *I* > 2σ(*I*)
Graphite monochromator	*R*_int_ = 0.054
phi and ω scans	θ_max_ = 28.1°, θ_min_ = 1.9°
Absorption correction: multi-scan (*SADABS*; Bruker, 2008)	*h* = −14→14
*T*_min_ = 0.520, *T*_max_ = 0.713	*k* = −17→17
31509 measured reflections	*l* = −20→20

### Refinement

**Table:**

Refinement on *F*^2^	Primary atom site location: structure-invariant direct methods
Least-squares matrix: full	Secondary atom site location: difference Fourier map
*R*[*F*^2^ > 2σ(*F*^2^)] = 0.032	Hydrogen site location: mixed
*wR*(*F*^2^) = 0.082	H-atom parameters constrained
*S* = 1.01	*w* = 1/[σ^2^(*F*_o_^2^) + (0.037*P*)^2^ + 1.2826*P*] where *P* = (*F*_o_^2^ + 2*F*_c_^2^)/3
5356 reflections	(Δ/σ)_max_ = 0.001
273 parameters	Δρ_max_ = 0.87 e Å^−^^3^
0 restraints	Δρ_min_ = −0.48 e Å^−^^3^

### Special details

**Table:**

Geometry. All s.u.'s (except the s.u.'s in the dihedral angle between two l.s. planes) are estimated using the full covariance matrix. The cell s.u.'s are taken into account individually in the estimation of s.u.'s in distances, angles and torsion angles; correlations between s.u.'s in cell parameters are only used when they are defined by crystal symmetry. An approximate (isotropic) treatment of cell s.u.'s is used for estimating s.u.'s involving l.s. planes.
Refinement. Refinement of *F*^2^ against ALL reflections. The weighted *R*-factor *wR* and goodness of fit *S* are based on *F*^2^, conventional *R*-factors *R* are based on *F*, with *F* set to zero for negative *F*^2^. The threshold expression of *F*^2^ > σ(*F*^2^) is used only for calculating *R*-factors(gt) *etc*. and is not relevant to the choice of reflections for refinement. *R*-factors based on *F*^2^ are statistically about twice as large as those based on *F*, and *R*- factors based on ALL data will be even larger.

### Fractional atomic coordinates and isotropic or equivalent isotropic displacement parameters (Å^2^)

**Table:**

	*x*	*y*	*z*	*U*_iso_*/*U*_eq_	
Br1	0.543412 (18)	0.702949 (15)	0.181863 (12)	0.02186 (7)	
O1	0.83014 (13)	0.48942 (12)	0.72034 (9)	0.0258 (3)	
N1	0.62069 (14)	0.47059 (12)	0.61743 (10)	0.0168 (3)	
N2	0.67921 (15)	0.46201 (12)	0.48350 (10)	0.0192 (3)	
N3	0.68765 (14)	0.36830 (12)	0.52479 (10)	0.0178 (3)	
N4	0.64780 (15)	0.29581 (12)	0.65761 (10)	0.0204 (3)	
H3N	0.5982	0.2991	0.7008	0.025*	
N5	0.62162 (16)	0.62146 (13)	0.53199 (11)	0.0229 (4)	
H1N	0.6464	0.6474	0.4831	0.028*	
H2N	0.6076	0.6534	0.5810	0.028*	
C1	0.65153 (17)	0.37328 (14)	0.60340 (12)	0.0174 (4)	
C2	0.64195 (17)	0.52227 (15)	0.54245 (12)	0.0175 (4)	
C3	0.72309 (18)	0.27569 (14)	0.48255 (12)	0.0190 (4)	
H3A	0.6502	0.2511	0.4432	0.023*	
C4	0.75412 (19)	0.19832 (15)	0.55517 (13)	0.0221 (4)	
H4A	0.7612	0.1310	0.5292	0.027*	
H4B	0.8363	0.2152	0.5864	0.027*	
C5	0.65589 (19)	0.19465 (14)	0.61958 (13)	0.0219 (4)	
H5A	0.5730	0.1765	0.5885	0.026*	
C6	0.6897 (2)	0.11877 (16)	0.69099 (14)	0.0282 (5)	
H6A	0.6306	0.1245	0.7348	0.042*	
H6B	0.7746	0.1320	0.7175	0.042*	
H6C	0.6856	0.0509	0.6667	0.042*	
C7	0.83408 (17)	0.28950 (14)	0.43140 (12)	0.0180 (4)	
C8	0.92976 (18)	0.35757 (16)	0.45378 (13)	0.0235 (4)	
H8A	0.9239	0.4023	0.5007	0.028*	
C9	1.0336 (2)	0.36042 (17)	0.40785 (14)	0.0281 (5)	
H9A	1.0979	0.4080	0.4229	0.034*	
C10	1.0448 (2)	0.29453 (17)	0.34016 (14)	0.0295 (5)	
H10A	1.1168	0.2963	0.3095	0.035*	
C11	0.9504 (2)	0.22620 (18)	0.31757 (13)	0.0298 (5)	
H11A	0.9574	0.1806	0.2715	0.036*	
C12	0.8450 (2)	0.22454 (16)	0.36260 (13)	0.0240 (4)	
H12A	0.7796	0.1784	0.3462	0.029*	
C13	0.60915 (17)	0.51128 (15)	0.70338 (11)	0.0181 (4)	
H13A	0.5505	0.4696	0.7332	0.022*	
H13B	0.5755	0.5804	0.6985	0.022*	
C14	0.73822 (18)	0.51207 (14)	0.75576 (12)	0.0188 (4)	
C15	0.74642 (18)	0.54203 (14)	0.84815 (12)	0.0194 (4)	
C16	0.8639 (2)	0.56537 (17)	0.88979 (14)	0.0275 (4)	
H16A	0.9360	0.5606	0.8594	0.033*	
C17	0.8757 (2)	0.59558 (18)	0.97557 (14)	0.0316 (5)	
H17A	0.9557	0.6123	1.0034	0.038*	
C18	0.7711 (2)	0.60135 (17)	1.02054 (13)	0.0285 (5)	
H18A	0.7794	0.6226	1.0790	0.034*	
C19	0.6546 (2)	0.57634 (16)	0.98056 (13)	0.0259 (4)	
H19A	0.5833	0.5793	1.0119	0.031*	
C20	0.64158 (19)	0.54670 (15)	0.89427 (12)	0.0222 (4)	
H20A	0.5614	0.5297	0.8669	0.027*	
O1S	0.69870 (16)	0.69468 (12)	0.37452 (10)	0.0329 (4)	
H1S	0.6682	0.7057	0.3229	0.049*	
C1S	0.8245 (2)	0.6690 (2)	0.36528 (16)	0.0407 (6)	
H1SA	0.8698	0.6601	0.4233	0.049*	
H1SB	0.8644	0.7252	0.3368	0.049*	
C2S	0.8375 (2)	0.57495 (19)	0.31379 (17)	0.0395 (6)	
H2SA	0.9263	0.5579	0.3141	0.059*	
H2SB	0.8013	0.5858	0.2543	0.059*	
H2SC	0.7936	0.5198	0.3394	0.059*	

### Atomic displacement parameters (Å^2^)

**Table:**

	*U*^11^	*U*^22^	*U*^33^	*U*^12^	*U*^13^	*U*^23^
Br1	0.02512 (11)	0.02392 (11)	0.01705 (10)	−0.00020 (8)	0.00457 (7)	0.00345 (8)
O1	0.0210 (7)	0.0330 (8)	0.0241 (7)	0.0005 (6)	0.0060 (6)	−0.0020 (6)
N1	0.0212 (8)	0.0161 (8)	0.0135 (7)	0.0028 (6)	0.0038 (6)	−0.0005 (6)
N2	0.0223 (8)	0.0176 (8)	0.0180 (8)	0.0019 (6)	0.0032 (6)	0.0014 (6)
N3	0.0200 (8)	0.0176 (8)	0.0163 (7)	0.0023 (6)	0.0033 (6)	−0.0010 (6)
N4	0.0258 (8)	0.0176 (8)	0.0190 (8)	0.0014 (6)	0.0078 (6)	0.0015 (6)
N5	0.0322 (9)	0.0194 (9)	0.0185 (8)	0.0042 (7)	0.0095 (7)	0.0023 (6)
C1	0.0155 (9)	0.0189 (9)	0.0178 (9)	−0.0002 (7)	0.0020 (7)	−0.0007 (7)
C2	0.0161 (9)	0.0217 (10)	0.0148 (8)	0.0001 (7)	0.0022 (7)	0.0015 (7)
C3	0.0201 (9)	0.0192 (10)	0.0179 (9)	−0.0008 (7)	0.0034 (7)	−0.0055 (7)
C4	0.0256 (10)	0.0162 (9)	0.0255 (10)	−0.0006 (8)	0.0069 (8)	−0.0025 (8)
C5	0.0241 (10)	0.0177 (10)	0.0246 (10)	−0.0023 (8)	0.0060 (8)	−0.0015 (8)
C6	0.0381 (12)	0.0191 (10)	0.0285 (11)	−0.0004 (9)	0.0098 (9)	0.0021 (8)
C7	0.0186 (9)	0.0184 (9)	0.0170 (8)	0.0026 (7)	0.0023 (7)	−0.0001 (7)
C8	0.0233 (10)	0.0229 (10)	0.0244 (10)	−0.0003 (8)	0.0027 (8)	−0.0037 (8)
C9	0.0235 (10)	0.0262 (11)	0.0348 (12)	−0.0026 (8)	0.0042 (9)	0.0020 (9)
C10	0.0276 (11)	0.0338 (12)	0.0291 (11)	0.0055 (9)	0.0132 (9)	0.0063 (9)
C11	0.0357 (12)	0.0328 (12)	0.0219 (10)	0.0047 (9)	0.0091 (9)	−0.0056 (9)
C12	0.0265 (10)	0.0232 (10)	0.0223 (10)	−0.0001 (8)	0.0032 (8)	−0.0036 (8)
C13	0.0220 (9)	0.0193 (9)	0.0140 (8)	0.0027 (7)	0.0064 (7)	−0.0005 (7)
C14	0.0227 (10)	0.0148 (9)	0.0194 (9)	−0.0014 (7)	0.0044 (7)	0.0015 (7)
C15	0.0249 (10)	0.0157 (9)	0.0171 (9)	0.0010 (7)	−0.0003 (7)	0.0017 (7)
C16	0.0253 (11)	0.0288 (11)	0.0282 (11)	−0.0008 (8)	0.0023 (8)	−0.0024 (9)
C17	0.0310 (12)	0.0350 (13)	0.0269 (11)	−0.0038 (9)	−0.0070 (9)	−0.0023 (9)
C18	0.0414 (13)	0.0245 (11)	0.0185 (10)	0.0009 (9)	−0.0027 (8)	−0.0022 (8)
C19	0.0335 (11)	0.0231 (10)	0.0214 (10)	0.0020 (8)	0.0052 (8)	0.0009 (8)
C20	0.0258 (10)	0.0212 (10)	0.0195 (9)	0.0013 (8)	0.0013 (7)	−0.0006 (8)
O1S	0.0432 (9)	0.0366 (9)	0.0191 (7)	0.0049 (7)	0.0041 (6)	0.0034 (6)
C1S	0.0407 (14)	0.0546 (16)	0.0265 (12)	−0.0076 (12)	0.0015 (10)	−0.0021 (11)
C2S	0.0408 (14)	0.0338 (13)	0.0462 (14)	0.0064 (10)	0.0156 (11)	0.0128 (11)

### Geometric parameters (Å, º)

**Table:**

O1—C14	1.217 (2)	C9—H9A	0.9500
N1—C1	1.361 (2)	C10—C11	1.382 (3)
N1—C2	1.394 (2)	C10—H10A	0.9500
N1—C13	1.461 (2)	C11—C12	1.390 (3)
N2—C2	1.312 (2)	C11—H11A	0.9500
N2—N3	1.403 (2)	C12—H12A	0.9500
N3—C1	1.323 (2)	C13—C14	1.537 (3)
N3—C3	1.467 (2)	C13—H13A	0.9900
N4—C1	1.337 (2)	C13—H13B	0.9900
N4—C5	1.479 (2)	C14—C15	1.488 (3)
N4—H3N	0.9001	C15—C16	1.396 (3)
N5—C2	1.347 (2)	C15—C20	1.397 (3)
N5—H1N	0.9000	C16—C17	1.389 (3)
N5—H2N	0.9001	C16—H16A	0.9500
C3—C7	1.510 (3)	C17—C18	1.385 (3)
C3—C4	1.542 (3)	C17—H17A	0.9500
C3—H3A	1.0000	C18—C19	1.382 (3)
C4—C5	1.528 (3)	C18—H18A	0.9500
C4—H4A	0.9900	C19—C20	1.395 (3)
C4—H4B	0.9900	C19—H19A	0.9500
C5—C6	1.520 (3)	C20—H20A	0.9500
C5—H5A	1.0000	O1S—C1S	1.416 (3)
C6—H6A	0.9800	O1S—H1S	0.8502
C6—H6B	0.9800	C1S—C2S	1.503 (4)
C6—H6C	0.9800	C1S—H1SA	0.9900
C7—C8	1.389 (3)	C1S—H1SB	0.9900
C7—C12	1.392 (3)	C2S—H2SA	0.9800
C8—C9	1.385 (3)	C2S—H2SB	0.9800
C8—H8A	0.9500	C2S—H2SC	0.9800
C9—C10	1.387 (3)		
			
C1—N1—C2	105.84 (14)	C10—C9—H9A	119.6
C1—N1—C13	123.01 (15)	C11—C10—C9	119.59 (19)
C2—N1—C13	128.34 (16)	C11—C10—H10A	120.2
C2—N2—N3	103.54 (14)	C9—C10—H10A	120.2
C1—N3—N2	111.60 (15)	C10—C11—C12	119.7 (2)
C1—N3—C3	124.82 (16)	C10—C11—H11A	120.1
N2—N3—C3	123.43 (15)	C12—C11—H11A	120.1
C1—N4—C5	116.37 (16)	C11—C12—C7	121.0 (2)
C1—N4—H3N	119.5	C11—C12—H12A	119.5
C5—N4—H3N	114.0	C7—C12—H12A	119.5
C2—N5—H1N	114.9	N1—C13—C14	109.53 (14)
C2—N5—H2N	113.6	N1—C13—H13A	109.8
H1N—N5—H2N	128.6	C14—C13—H13A	109.8
N3—C1—N4	125.18 (17)	N1—C13—H13B	109.8
N3—C1—N1	107.16 (16)	C14—C13—H13B	109.8
N4—C1—N1	127.66 (17)	H13A—C13—H13B	108.2
N2—C2—N5	124.96 (17)	O1—C14—C15	122.21 (18)
N2—C2—N1	111.78 (16)	O1—C14—C13	119.20 (17)
N5—C2—N1	123.22 (16)	C15—C14—C13	118.58 (16)
N3—C3—C7	112.85 (16)	C16—C15—C20	119.32 (18)
N3—C3—C4	106.27 (15)	C16—C15—C14	118.22 (17)
C7—C3—C4	110.14 (16)	C20—C15—C14	122.45 (18)
N3—C3—H3A	109.2	C17—C16—C15	120.2 (2)
C7—C3—H3A	109.2	C17—C16—H16A	119.9
C4—C3—H3A	109.2	C15—C16—H16A	119.9
C5—C4—C3	112.97 (16)	C18—C17—C16	120.1 (2)
C5—C4—H4A	109.0	C18—C17—H17A	119.9
C3—C4—H4A	109.0	C16—C17—H17A	119.9
C5—C4—H4B	109.0	C19—C18—C17	120.2 (2)
C3—C4—H4B	109.0	C19—C18—H18A	119.9
H4A—C4—H4B	107.8	C17—C18—H18A	119.9
N4—C5—C6	109.42 (16)	C18—C19—C20	120.15 (19)
N4—C5—C4	107.82 (15)	C18—C19—H19A	119.9
C6—C5—C4	111.81 (17)	C20—C19—H19A	119.9
N4—C5—H5A	109.3	C19—C20—C15	119.98 (19)
C6—C5—H5A	109.2	C19—C20—H20A	120.0
C4—C5—H5A	109.2	C15—C20—H20A	120.0
C5—C6—H6A	109.5	C1S—O1S—H1S	103.1
C5—C6—H6B	109.5	O1S—C1S—C2S	113.4 (2)
H6A—C6—H6B	109.5	O1S—C1S—H1SA	108.9
C5—C6—H6C	109.5	C2S—C1S—H1SA	108.9
H6A—C6—H6C	109.5	O1S—C1S—H1SB	108.9
H6B—C6—H6C	109.5	C2S—C1S—H1SB	108.9
C8—C7—C12	118.81 (18)	H1SA—C1S—H1SB	107.7
C8—C7—C3	123.43 (17)	C1S—C2S—H2SA	109.5
C12—C7—C3	117.55 (17)	C1S—C2S—H2SB	109.5
C9—C8—C7	120.17 (19)	H2SA—C2S—H2SB	109.5
C9—C8—H8A	119.9	C1S—C2S—H2SC	109.5
C7—C8—H8A	119.9	H2SA—C2S—H2SC	109.5
C8—C9—C10	120.7 (2)	H2SB—C2S—H2SC	109.5
C8—C9—H9A	119.6		
			
C2—N2—N3—C1	2.6 (2)	N3—C3—C7—C8	31.3 (3)
C2—N2—N3—C3	178.23 (16)	C4—C3—C7—C8	−87.2 (2)
N2—N3—C1—N4	179.06 (17)	N3—C3—C7—C12	−154.10 (17)
C3—N3—C1—N4	3.5 (3)	C4—C3—C7—C12	87.3 (2)
N2—N3—C1—N1	−1.3 (2)	C12—C7—C8—C9	0.2 (3)
C3—N3—C1—N1	−176.87 (16)	C3—C7—C8—C9	174.75 (19)
C5—N4—C1—N3	−15.1 (3)	C7—C8—C9—C10	−1.2 (3)
C5—N4—C1—N1	165.38 (18)	C8—C9—C10—C11	0.9 (3)
C2—N1—C1—N3	−0.5 (2)	C9—C10—C11—C12	0.3 (3)
C13—N1—C1—N3	−162.89 (16)	C10—C11—C12—C7	−1.2 (3)
C2—N1—C1—N4	179.17 (18)	C8—C7—C12—C11	1.0 (3)
C13—N1—C1—N4	16.7 (3)	C3—C7—C12—C11	−173.87 (19)
N3—N2—C2—N5	179.50 (18)	C1—N1—C13—C14	66.8 (2)
N3—N2—C2—N1	−2.8 (2)	C2—N1—C13—C14	−91.5 (2)
C1—N1—C2—N2	2.2 (2)	N1—C13—C14—O1	6.3 (2)
C13—N1—C2—N2	163.37 (17)	N1—C13—C14—C15	−174.53 (15)
C1—N1—C2—N5	179.90 (18)	O1—C14—C15—C16	13.9 (3)
C13—N1—C2—N5	−18.9 (3)	C13—C14—C15—C16	−165.25 (18)
C1—N3—C3—C7	−140.15 (18)	O1—C14—C15—C20	−165.57 (19)
N2—N3—C3—C7	44.8 (2)	C13—C14—C15—C20	15.3 (3)
C1—N3—C3—C4	−19.3 (2)	C20—C15—C16—C17	−1.8 (3)
N2—N3—C3—C4	165.59 (16)	C14—C15—C16—C17	178.8 (2)
N3—C3—C4—C5	46.9 (2)	C15—C16—C17—C18	0.9 (3)
C7—C3—C4—C5	169.40 (16)	C16—C17—C18—C19	0.5 (3)
C1—N4—C5—C6	163.01 (17)	C17—C18—C19—C20	−1.0 (3)
C1—N4—C5—C4	41.2 (2)	C18—C19—C20—C15	0.2 (3)
C3—C4—C5—N4	−59.0 (2)	C16—C15—C20—C19	1.2 (3)
C3—C4—C5—C6	−179.28 (17)	C14—C15—C20—C19	−179.30 (19)

### Hydrogen-bond geometry (Å, º)

**Table:**

*D*—H···*A*	*D*—H	H···*A*	*D*···*A*	*D*—H···*A*
N4—H3*N*···Br1^i^	0.90	2.49	3.392 (2)	176
N5—H1*N*···O1*S*	0.90	1.94	2.839 (2)	176
N5—H2*N*···Br1^ii^	0.90	2.61	3.468 (2)	159
O1*S*—H1*S*···Br1	0.85	2.46	3.287 (2)	165

Symmetry codes: (i) −*x*+1, −*y*+1, −*z*+1; (ii) *x*, −*y*+3/2, *z*+1/2.
